# The role of the globular heads of the C1q receptor in TcdA-induced human colonic epithelial cell apoptosis via a mitochondria-dependent pathway

**DOI:** 10.1186/s12866-020-01958-6

**Published:** 2020-09-02

**Authors:** Jinhua Liang, Yongzhong Ning, Li Dong, Xiufeng Ma, Shu Li, Heran Yang, Qi Li, Ling Chen, Lingjuan Gao, Yanmin Xu

**Affiliations:** 1 Department of Clinical Laboratory, Hongqi Hospital Affiliated to Mudanjiang Medical University, Mudanjiang, China; 2 Department of Clinical Laboratory, Beijing Chuiyangliu Hospital Affiliated to Tsinghua University, Beijing, China; 3 Three Department of General Surgery, Hongqi Hospital Affiliated to Mudanjiang Medical College, Tongxiang Road, Aimin District, Mudanjiang, 157000 China; 4 Department of Clinical Laboratory, Lishui District Maternal and Child Health Care Center, Nanjing, China; 5 Institute of translational medicine, Nanjing medical university, Nanjing, China

**Keywords:** *Clostridium difficile*, TcdA, gC1qR, Mitochondrial function, apoptosis

## Abstract

**Background:**

*Clostridioides* (formerly *Clostridium) difficile* infection is the leading cause of antibiotic-associated colitis. Studies have demonstrated that *C. difficile* toxin A (TcdA) can cause apoptosis of many human cell types. The purpose of this study was to investigate the relationships among exposure to TcdA, the role of the receptor for the globular heads of C1q (gC1qR) gene and the underlying intracellular apoptotic mechanism in human colonic epithelial cells (NCM 460). In this study, gC1qR expression was examined using real-time polymerase chain reaction (PCR), western blotting and immunohistochemical staining. Cell viability was assessed by the water-soluble tetrazolium salt (WST-1) assay, and cell apoptosis was assessed by flow cytometry and the terminal deoxynucleotidyl transferase-mediated deoxyuridine triphosphate nick-end labeling (TUNEL) assay. Mitochondrial function was assessed based on reactive oxygen species (ROS) generation, changes in the mitochondrial membrane potential (ΔΨm) and the content of ATP.

**Results:**

Our study demonstrated that increasing the concentration of TcdA from 10 ng/ml to 20 ng/ml inhibited cell viability and induced cell apoptosis (*p* < 0.01). Moreover, the TcdA-induced gC1qR expression and enhanced expression of gC1qR caused mitochondrial dysfunction (including production of ROS and decreases in the ΔΨm and the content of ATP) and cell apoptosis. However, silencing of the gC1qR gene reversed TcdA-induced cell apoptosis and mitochondrial dysfunction.

**Conclusion:**

These data support a mechanism by which gC1qR plays a crucial role in TcdA-induced apoptosis of human colonic epithelial cells in a mitochondria-dependent manner.

## Background

*Clostridium difficile* is now referred to as *Clostridioides difficile* (*C. difficile*). *C. difficile* infection (CDI) is the leading cause of antibiotic-associated colitis, a disease with high morbidity and mortality that imposes a major economic burden on hospitalized patients. In recent years, the incidence of CDI has been significantly increasing, and CDI is becoming the main cause of hospital infections in developed countries [1–3]. *C. difficile* causes intestinal damage and diarrhea mainly because it produces two key virulence determinants, toxin A and toxin B, in the intestinal lumen [4, 5]. TcdA was shown to produce an intense inflammatory response, including mucosal disruption, mast cell degranulation, fluid accumulation, epithelial cell death, edema, and severe neutrophil infiltration [6–9]. In vitro studies have demonstrated that TcdA can cause apoptosis of many human cell types, including monocytes [10], HeLa cells [11], endothelial cells [12], and intestinal epithelial cells [13–15]. The mechanisms of TcdA-induced apoptosis remain to be fully characterized. Zhang et al. demonstrated that toxin A treatment results in a significant loss of viability and apoptosis in a neuronal cell line; this effect was found to depend on increased production of reactive oxygen species (ROS) and upregulation of p38 MAPK activity and p21^Cip1/Waf1^ expression [16]. Carneiro et al. have demonstrated that TcdA induces the cleavage of caspases 6, 8, 9, and 3 and Bid leading to human intestinal epithelial cell death [17]. However, the role of mitochondrial dysfunction in TcdA-induced human intestinal epithelial cell death has not been thoroughly investigated.

Complement activation is an important characteristic of cytotoxic injury. In the process of complement activation, C1q can act as a powerful extracellular signal for a wide range of cells, leading to the ligand-specific biological responses [18]. These responses are generally mediated by two cell surface molecules, receptor for the globular heads of C1q (gC1qR) and receptor for the collagen tail of C1q (cC1qR). gC1qR is a ubiquitous cellular protein with high anionic properties, which was initially identified as a protein of the mitochondrial matrix [19]. There is evidence that gC1qR mediates a number of biological responses including infection, inflammation, and immune regulation. Examples of these reactions include growth disturbances, morphological abnormalities, and the initiation of apoptosis [20].

In this study, we explored the mechanism of TcdA-induced cell apoptosis using human colonic NCM 460 epithelial cell lines. We demonstrate that TcdA-induced cell death strongly dependent on the induction of the mitochondrial dysfunction pathway. Therefore, we provide evidence that gC1qR gene plays an important role in TcdA-induced epithelial cell apoptosis involving mitochondrial dependent manner.

## Results

### *C. difficile* TcdA induces cell apoptosis in human colonic epithelial cells

**Fig. 1:**
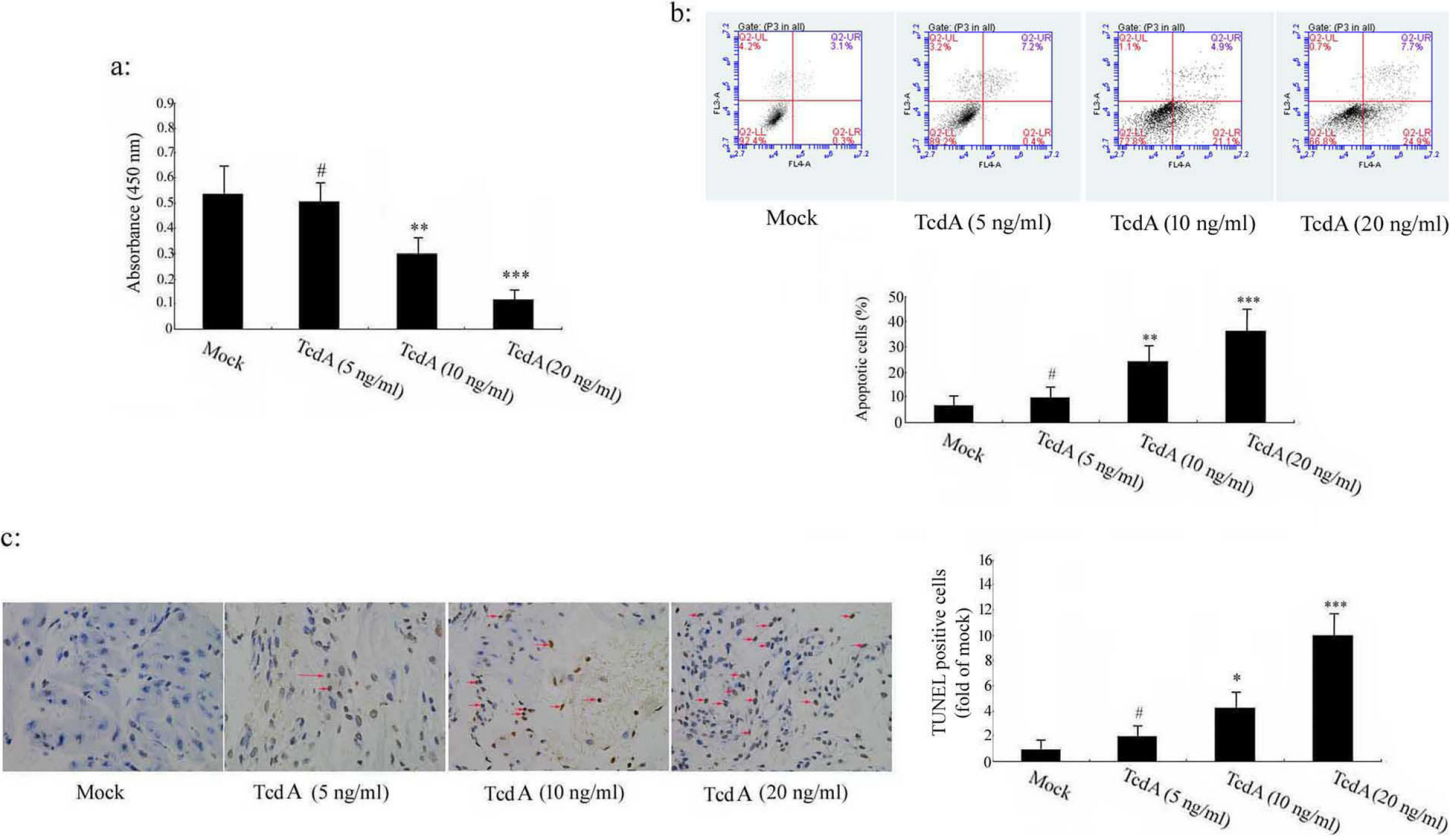
Sensitivity of human colonic epithelial cell to TcdA-induced cell apoptosis. The human colonic epithelial cells were exposed to 5 ng/ml, 10 ng/ml, 20 ng/ml TcdA for 24 h. **a** The viability of cells was detected by WST-1 assay (*n* = 3 individual experiments). **b** Apoptotic death of NCM 460 cells was assessed by flow cytometric analysis (n = 3). Q2_LL represents normal cells, and the early and late apoptotic cells are located in the Q2_LR and Q2_UR regions. The necrotic cells are distributed in the Q2_UL region. The relative ratio of early and lately apoptotic cells was chosen for further comparison. **c** TUNNEL staining of human colonic epithelial cells. Cells with shrunken brown stained nuclei were considered positive (Red arrows). ***p < 0.001, **p < 0.01, *p < 0.05, ^#^*p* > 0.05 versus Mock group (Control group)

In this study, we used the human colonic NCM 460 epithelial cell lines as model to investigate the mechanisms of TcdA-induced cell apoptosis. NCM 460 cells were treated with different concentrations of TcdA (5 ng/ml, 10 ng/ml, 20 ng/ml). The results demonstrated that TcdA at 5 ng/ml treatment slightly decreased cell viability and increased cell apoptosis, but did not reach a statistically significant level (*p* > 0.05). In contrast, the concentration of TcdA increasing from 10 ng/ml to 20 ng/ml significantly decreased cell viability and apparently increased cell apoptosis when compared with the mock group (*p* < 0.01, *p* < 0.001,). Quantification of the data indicated that cell viability decreased (Fig. 1a) and cell apoptosis (Fig. 1b-c) increased in a dose-dependent manner following TcdA treatment.

### The relationship between TcdA and gC1qR gene in human colonic NCM 460 epithelial cells

**Fig. 2:**
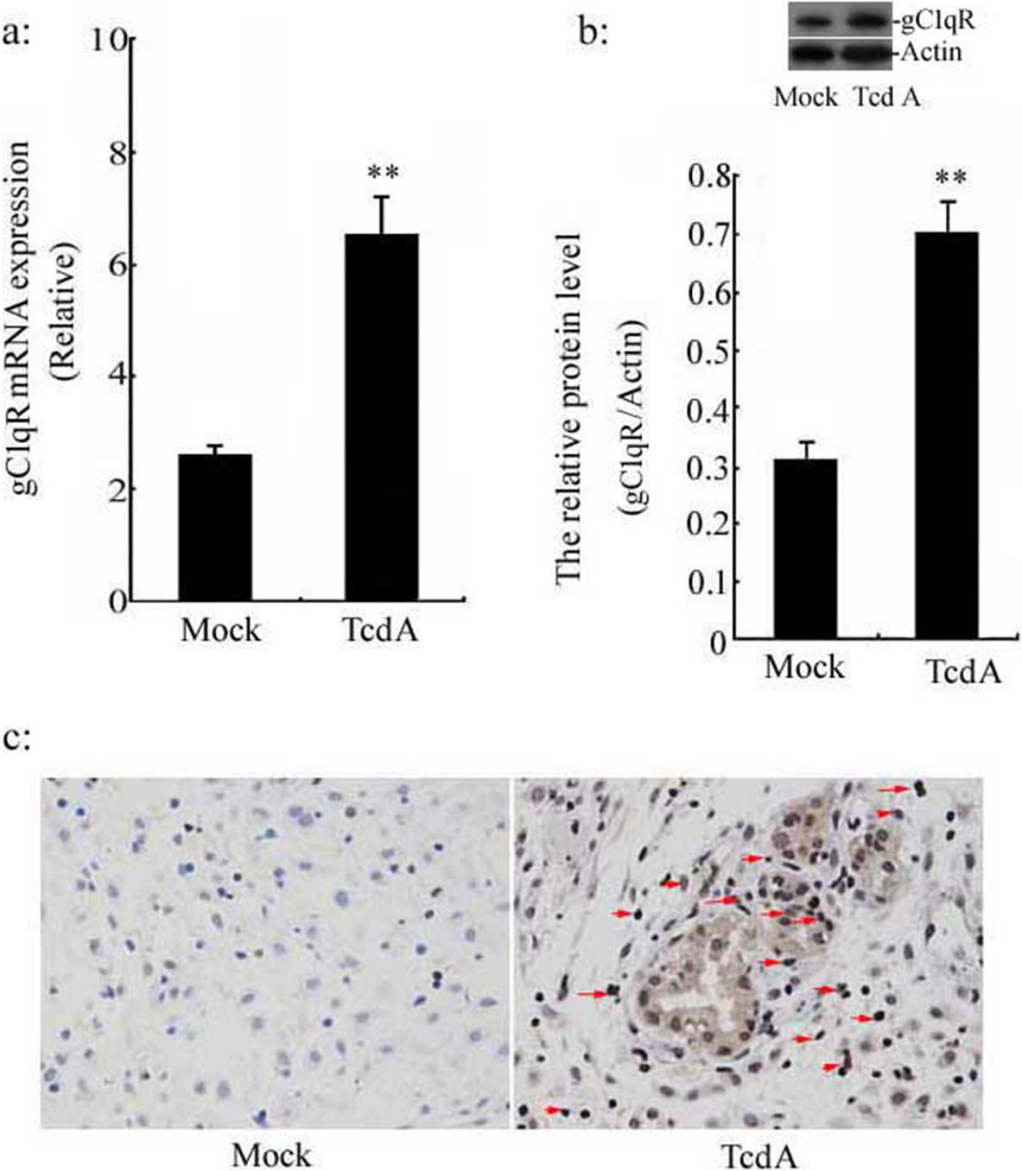
gC1qR expression in human colonic epithelial cell lines. The human colonic epithelial cells were treated with TcdA at 10 ng/ml for 24 h. **a** gC1qR mRNA level was detected using qRT-PCR; **b** The expression of gC1qR protein in lysates of NCM460 cells was measured by western blot assay. **c** Localization and expression of gC1qR protein was examined using immunohistochemical staining analysis. Cells with brown stained cytoplasm were considered positive (Red arrows). **p < 0.01 versus Mock group

Many features point to gC1qR localizes to the mitochondrial matrix, which mediates morphological abnormalities, growth perturbations and the initiation of apoptosis. To clarify whether the gC1qR gene is required for TcdA to entry into cells and induce apoptosis, firstly, we measured the gC1qR expression in the presence of TcdA at 10 ng/ml, the mRNA and protein expression of gC1qR were analyzed by qRT-PCR and western blot, respectively. The data revealed that gC1qR expression in the treatment TcdA group were significantly increased in NCM 460 cells compared with the mock group (Fig. 2a and b). Meanwhile, immunohistochemical staining analysis showed that gC1qR expression was apparently enhanced in the cytoplasm of human colonic NCM 460 epithelial cells in the treatment TcdA group (Fig. 2c, the enhanced expression of gC1qR protein anchored on the mitochondrial membrane surface, see Supplementary Figure 3); Secondly, the cell immunofluorescence assay showed that when the gC1qR gene was silenced, gC1qR protein expression was reduced and intracellular TcdA levels were also significantly reduced (see Supplementary Figure 12, gC1qR protein: green fluorescence and TcdA: blue fluorescence), in addition, the intracellular TcdA level was also detected by western blot analysis, the data showed that when gC1qR gene was silenced, the intracellular TcdA level was also significantly reduced (see Supplementary Figure 14). Thirdly, a brightfield picture showing cells rounded up in TcdA treated-NCM 460 cells, however, when the gC1qR gene was silenced, the cells gather phenomenon was disappeared (see Supplementary Figure 13).

### Overexpression of gC1qR induced mitochondrial dysfunction in human colonic NCM 460 epithelial cell lines

**Fig. 3:**
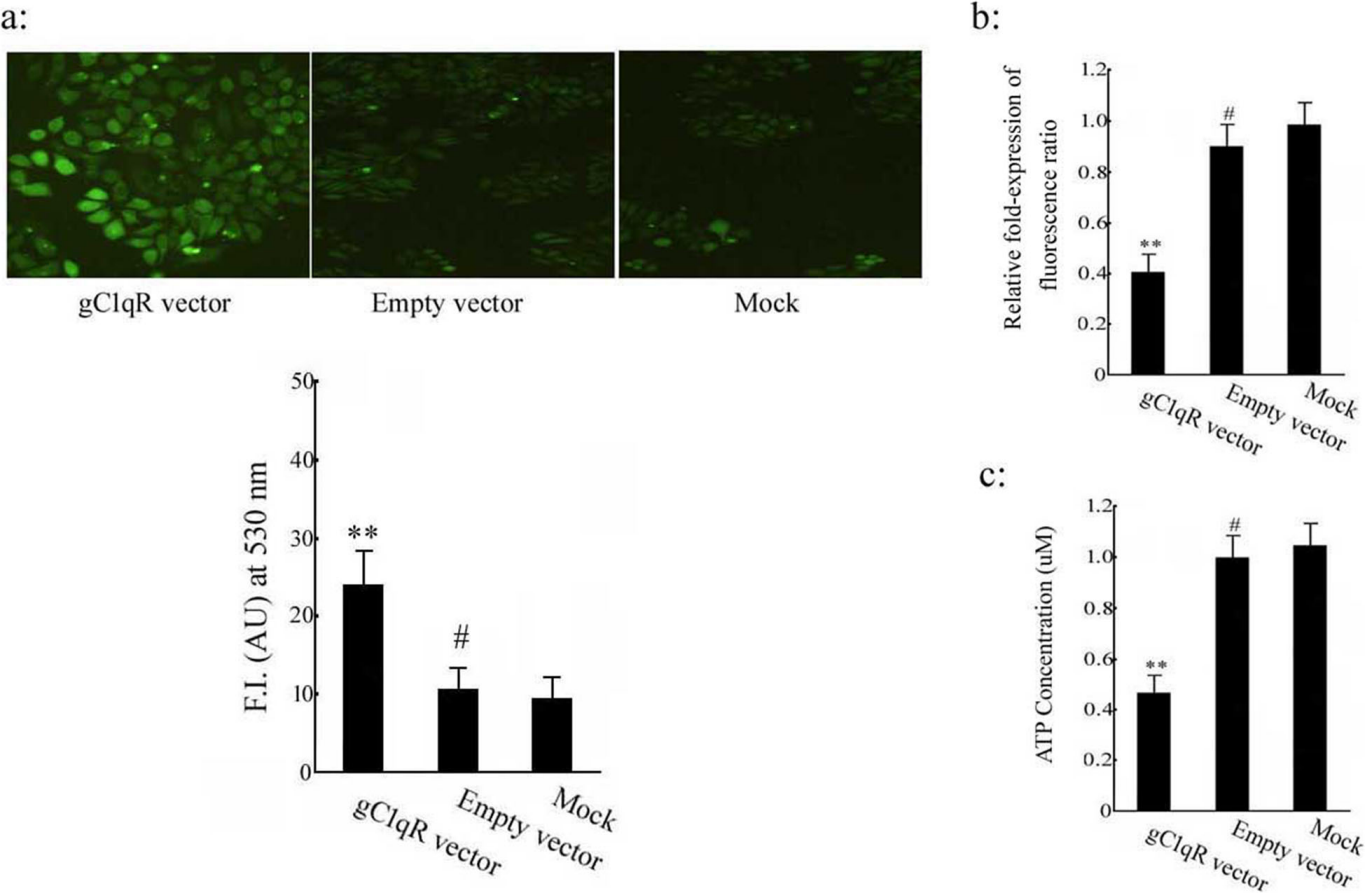
The effect of overexpression of gC1qR on mitochondrial function of human colonic NCM 460 epithelial cell lines. The human colonic epithelial cells were transfected with gC1qR vector or empty vector for 48 h. **a** Intracellular ROS generation was determined by fluorescence of H_2_DCFDA (Green). **b** The change of mitochondrial membrane potential was detected. The relative Δψm value was measured by monitoring the fluorescence of JC-1 (590: 527 nm fluorescence ratio). **c** ATP content was detected in NCM 460 epithelial cell. The data are means ± S.D. of three separate experiments performed in triplicate. **p < 0.01, ^#^*p* > 0.05 versus Mock group

The effect of the gC1qR gene on mitochondrial function in human colonic NCM 460 epithelial cells was explored in this study. To do this, we enhanced the gC1qR gene expression (gC1qR vector can effectively induce the expression of gC1qR protein, see Supplementary Figure 1), the ROS generation data showed that ROS levels in the gC1qR vector group were increased by approximately 2.67-fold compared with the mock group, but there was no difference between the mock group and empty vector group (Fig. 3a). Meanwhile, the effect of gC1qR on mitochondrial membrane potential was monitored by the uptake of JC-1 following transfection 48 h. The value of mitochondrial membrane potential in the gC1qR vector group decreased approximately 61.2% compared with the mock group (*p* < 0.01), indicating the mitochondrial membrane depolarization. There was no difference between the mock group and empty vector group (Fig. 3b). As shown in Fig. 3c, the accumulation of the gC1qR gene significantly decreased the content of ATP of NCM 460 cells when compared with mock group (*p* < 0.01), however, empty vector group and mock group, the mitochondrial ATP content showed no obvious changes.

### Effect of silencing of gC1qR gene on TcdA-induced cell apoptosis in human colonic epithelial cells

**Fig. 4:**
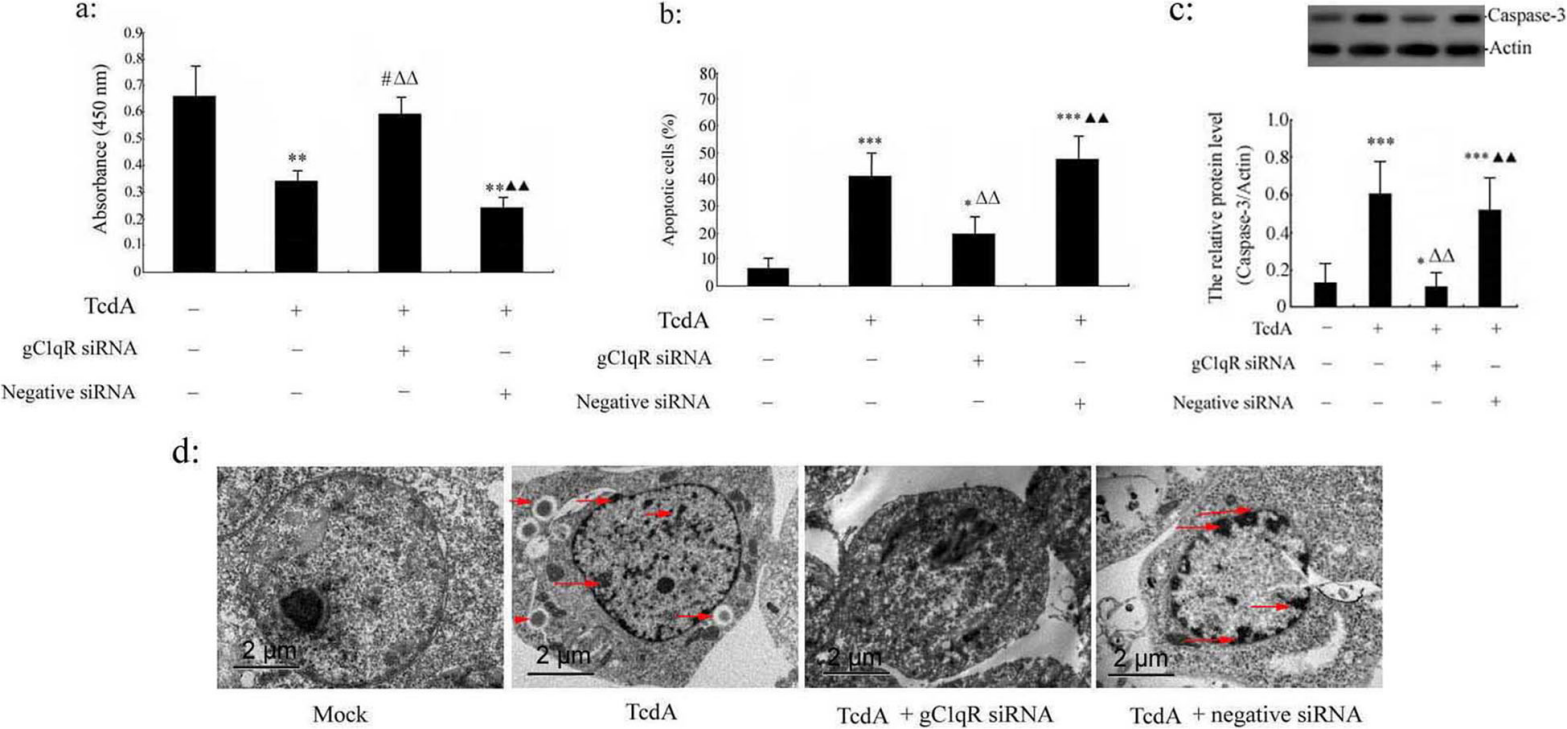
Effect of silencing of gC1qR gene on TcdA-induced cell apoptosis in human colonic epithelial cells. NCM 460 cells were transfected with gC1qR siRNA or negative siRNA for 48 h respectively, and then TcdA (10 ng/ml) was added for 24 h. **a** Cell viability was determined by WST-1 assay as described previously; **b** Apoptotic death of NCM 460 cells was examined by flow cytometric analysis. The data are means ± S.D. of three separate experiments performed in triplicate. **c** The expression of caspase-3 protein in lysates of NCM460 cells was measured by western blot assay. ***p < 0.001, **p < 0.01, *p < 0.05, ^#^*p* > 0.05 versus TcdA (−), gC1qR siRNA (−) and negative siRNA (−) group (Mock group); ^△△^*p* < 0.01 versus TcdA (+), gC1qR siRNA (−) and negative siRNA (−) group; ^▲▲^*p* < 0.01 versus TcdA (+), gC1qR siRNA (+) and negative siRNA (−) group. **d** The change of morphology of the NCM 460 cells was observed with an electron microscope. Red arrows point to the nuclei condensed and fractured, and chromatin increased and marginalized in the nucleus (3700 X)

To more completely understand the role of gC1qR in TcdA-induced cell apoptosis in human colonic NCM 460 epithelial cells, the cell viability and cells apoptosis were detected, respectively. TcdA significantly inhibited NCM 460 cells viability compared with the mock group (*p* < 0.01). Pretransfection with gC1qR siRNA (gC1qR siRNA can effectively silence the expression of gC1qR gene, see Supplementary Figure 2, and the silencing of gC1qR gene by siRNA was uniform in the human colonic epithelial cell, but not complete silencing of some cells and none in others, see Supplementary Figure 10) reversed TcdA-inhibited cell viability, as shown in Fig. 4a. The cell viability in the TcdA (+), gC1qR siRNA (+)-treated group significantly increased in comparison with those of the TcdA treatment alone group (*p* < 0.01), moreover, cell viability in the TcdA (+), negative siRNA (+) group significantly decreased compared with the TcdA (+), gC1qR siRNA (+) group (*p* < 0.01, Fig. 4a). Meanwhile, silence of gC1qR could alleviate the TcdA-induced cell apoptosis (Using rotenone (60 μM) (complex I inhibitor) or antimycine A (30 μM) (complex III inhibitor) could also alleviate the TcdA-induced cell apoptosis, see Supplementary Figure 5), the cell apoptosis in the TcdA (+), gC1qR siRNA (+)-treated group significantly decreased in comparison with those of the TcdA treatment alone group (*p* < 0.01), cell apoptosis in the TcdA (+), negative siRNA (+) group significantly increased compared with the TcdA (+), gC1qR siRNA (+) group (*p* < 0.01, Fig. 4b). In addition, our supplementary data show that either silencing of gC1qR gene or treatment with α-lipoic acid (α-lipoic acid was utilized in the current experiment, which has the properties of reducing ROS production and protecting mitochondrial function) could reverse TcdA-induced cell apoptosis (see Supplementary Figure 15). Next, the expression of apoptosis-related proteins, such as activated caspase-3 in NCM 460 cells were analyzed by Western blotting, the data revealed that the caspase-3 protein expression in the TcdA (+), gC1qR siRNA (+)-treated group significantly decreased in comparison with those of the TcdA treatment alone group (*p* < 0.01), moreover, compared with the TcdA (+), gC1qR siRNA (+) group, TcdA (+) and negative siRNA (+) group enhanced the expression of caspase-3 protein (*p* < 0.01, Fig. 4c and Supplementary Figure 8). The apoptotic morphology image of electron microscope was indicated in the TcdA (+) group and the TcdA (+), negative siRNA (+) group. These apoptotic morphology changes included nuclei condensed and fragmentation, chromatin condensation and marginalization, cell shrinkage, and apoptosis bodies consisting of the cytoplasm with tightly packed organelles (Fig. 4d and Supplementary Figure 9, red arrows).

### Effect of silencing of the gC1qR gene on TcdA-induced mitochondrial dysfunction in human colonic epithelial cells

**Fig. 5:**
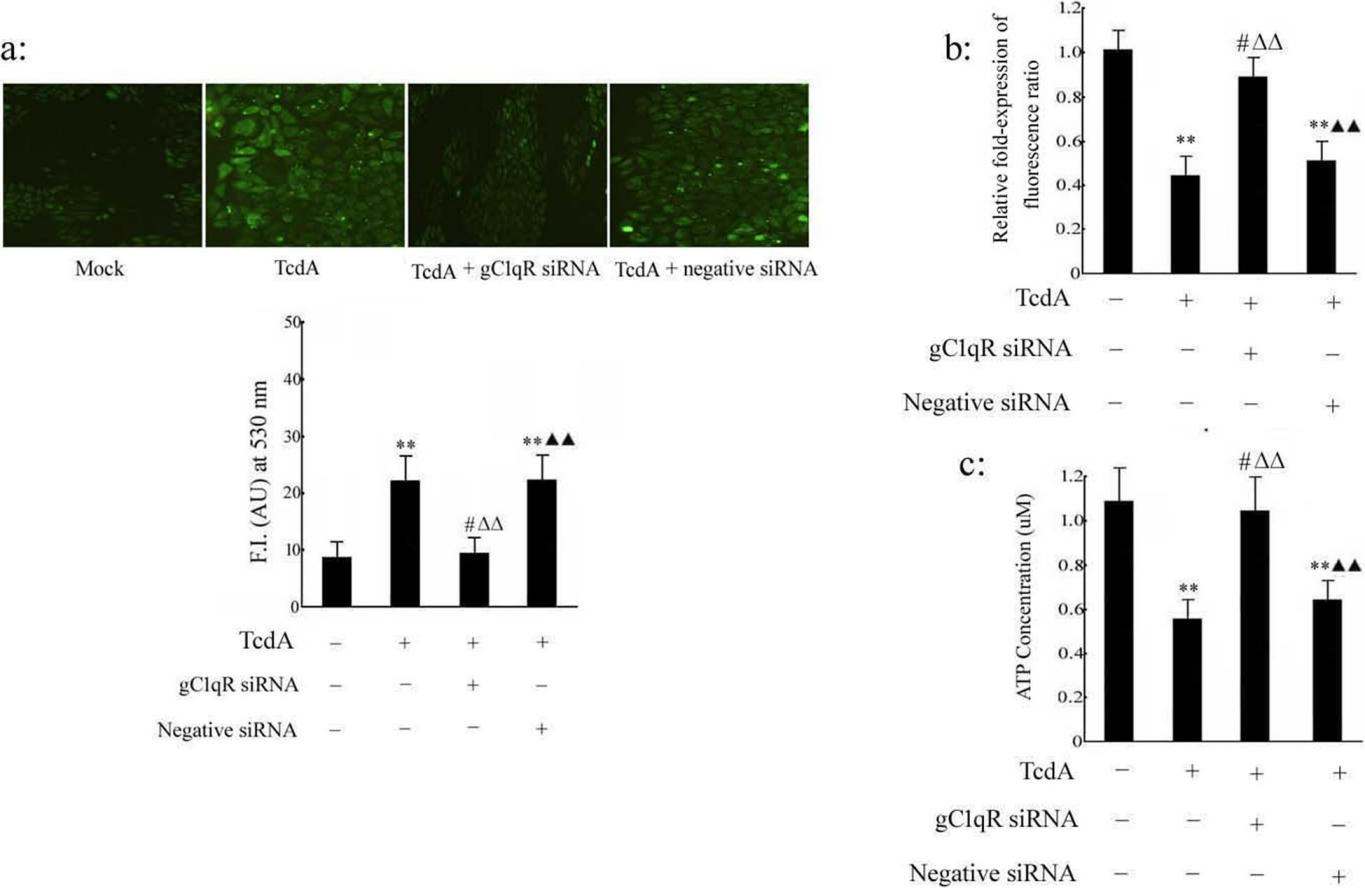
Effect of silencing of gC1qR gene on TcdA-induced mitochondrial dysfunction in human colonic epithelial cells. NCM 460 cells were transfected with gC1qR siRNA or negative siRNA for 48 h respectively, and then TcdA (10 ng/ml) was added for 24 h. **a** Intracellular ROS generation was measured by fluorescence of H_2_DCFDA (Green). **b** NCM 460 cells were stained with JC-1 and were subjected to flow cytometry. The relative Δψm value was examined by monitoring the fluorescence of JC-1 (590: 527 nm fluorescence ratio). **c** The ATP levels in NCM 460 epithelial cell were assessed. The data are presented as mean ± S.D. (*n* = 3). **p < 0.01, ^#^*p* > 0.05 versus TcdA (−), gC1qR siRNA (−) and negative siRNA (−) group; ^△△^*p* < 0.01versus TcdA (+), gC1qR siRNA (−) and negative siRNA (−) group; ^▲▲^*p* < 0.01 versus TcdA (+), gC1qR siRNA (+) and negative siRNA (−) group

In previous experiments, our data indicated that overexpression of the gC1qR gene can induce mitochondrial dysfunction (including the production of ROS and decreases in the mitochondrial membrane potential and the content of ATP). Compared with mock treatment, TcdA significantly enhanced ROS generation (*p* < 0.01). Pretransfection with gC1qR siRNA prevented TcdA-induced ROS accumulation; the levels of ROS in the TcdA (+), gC1qR siRNA (+)-treated group were significantly decreased compared with those in the TcdA alone-treated group (*p* < 0.01; silencing of gC1qR decreased the levels of mitochondrial respiratory chain NDUFS3 and core 2 protein levels. The mitochondrial respiratory chain is an important part of intracellular ROS production (see Supplementary Figure 4). Moreover, compared with TcdA (+), gC1qR siRNA (+) treatment, treatment with TcdA (+) and negative siRNA (+) caused a significant increase in ROS accumulation (*p* < 0.01, Fig. 5a). Silencing of the gC1qR gene attenuated the damage induced by TcdA. Mitochondrial dysfunction was manifested as decreases in the mitochondrial membrane potential and the content of ATP. We observed that the mitochondrial membrane potential and the content of ATP were lower in the TcdA group than in the mock group; however, gC1qR siRNA maintained the mitochondrial membrane potential and the content of ATP at normal levels in cells exposed to TcdA (Fig. 5b-c). The mitochondrial membrane potential and the content of ATP in the TcdA (+) and negative siRNA (+) group were significantly decreased compared to those in the TcdA (+) and gC1qR siRNA (+) group (*p* < 0.01; 0.6 mg/mL oligomycin was added to inhibit mitochondrial ATP synthase to confirm that the intracellular ATP was mitochondria-dependent ATP (see Supplementary Figure 6). PDH activity was also detected in TcdA-induced human colonic epithelial cells transfected with gC1qR siRNA. PDH activity was lower in the TcdA group than in the mock group; however, gC1qR siRNA maintained PDH activity at a normal level in cells exposed to TcdA (see Supplementary Figure 7).

## Discussion

It is known that *C. difficile* TcdA induces apoptosis of human epithelial cells in vitro; typical apoptotic changes include DNA fragmentation and caspase activation [21–23]. Apoptosis of human colonic epithelial cells caused by TcdA may play an important role in the pathogenicity of *C. difficile* in humans. In this study, we investigated the mechanism of TcdA-induced apoptosis of human colonic epithelial cells. We found that TcdA reduced the viability of human colonic epithelial cells and induced apoptosis in a dose-dependent manner. These findings are consistent with the current view that TcdA induces apoptosis of other epithelial cells. Our findings suggest that TcdA-induced apoptosis of human colonic epithelial cells is closely related to TcdA-induced expression of the gC1qR protein. In this process, TcdA required gC1qR as a receptor. Immunofluorescence laser confocal microscopy revealed a large number of C1q-gC1qR complexes anchored on the mitochondrial membrane surface in TcdA-induced human colonic epithelial cell. This is of importance, as gC1qR is required for TcdA entry into cells, one of the hallmarks is rounding up of cells. The cell immunofluorescence assay showed that the gC1qR gene was silenced, gC1qR protein expression was reduced and intracellular TcdA levels were also significantly reduced.

gC1qR is a multifunctional cellular protein expressed in a variety of tissues and cell types, including endothelial cells, dendritic cells, lymphocytes, and platelets [24]. As reported in the literature, by virtue of its ability to interact with a growing list of pathogen-associated molecules, including bacterial and viral ligands, gC1qR is gradually being recognized as an important pathogen recognition receptor (PRR). The major gC1qR-binding site of TcdA may be located in a highly conserved region encoded by exon IV and comprise residues 174–180. This conclusion is based on the fact that a wide range of bacterial and viral ligands are able to exploit gC1qR to suppress the host immune response and thus enhance the survival of the pathogens or enable them to gain access to the cells to induce disease [25] in addition to participating in regulating growth disturbances and initiating apoptosis [26]. In this study, we reported that apoptosis induced by TcdA is mediated by the gC1qR gene and found that the proapoptotic protein caspase-3 is involved in apoptosis. The data on apoptosis and iROS production are consistent with the effects of gC1qR gene silencing and the combination of the gC1qR gene silencing and TcdA treatment (see Supplementary Figure 11a-b).

The biological responses mediated by gC1qR are extensive; for example, gC1qR is involved in uptake, phagocytosis, and apoptosis in macrophages [27]. When constitutively expressed in a normal mouse fibroblast lines, gC1qR induces cell apoptosis [28]. gC1qR has been extensively studied, mainly as an induction factor of apoptosis. Chen reported that gC1qR induces apoptosis in cervical squamous cell carcinoma through the mitochondrial and p53-dependent pathways [29]. In this study, we found that gC1qR overexpression in human colonic epithelial cells led to an increasing in the rate of apoptosis. Recent cohort studies have shown that gC1qR is a conserved eukaryotic multifunctional protein mainly located in the mitochondrial matrix and at the cell surface. Human gC1qR is a 282-amino acid proprotein, and the first 73 amino acids contain the mitochondrial localization signal, which is necessary for localization of the protein in mitochondria; subsequently, the protein is cleaved to generate mature gC1qR [30, 31]. The mature form of gC1qR is associated with apoptosis and autophagy through induction of mitochondrial dysfunction [32]. There is growing evidence that mitochondrial dysfunction is linked to apoptosis induced by cytotoxic factors, such as ROS, which are overproduced in defective mitochondria. These findings raise questions about the role of gC1qR-induced mitochondrial dysfunction. Our study indicates that gC1qR overexpression in NCM 460 cells leads to the production of ROS and that continuous accumulation of ROS is related to a loss of ATP, which in turn disrupts the integrity of the mitochondrial membrane resulting in mitochondrial dysfunction and leading to apoptosis in colonic epithelial cells. These data support that either silencing of gC1qR gene or treatment with α-lipoic acid could reverse TcdA-induced cell apoptosis. Therefore, we promulgated to address the role of gC1qR gene in TcdA-induced NCM 460 cells apoptosis via a mitochondria-dependent pathway.

## Conclusion

In this report, we demonstrated that gC1qR expression is necessary in TcdA induced apoptosis of NCM460 cells. The silencing of gC1qR gene can protect mitochondrial function, including reducing ROS production, increasing ATP levels, and restoring mitochondrial membrane potential, thereby inhibiting NCM460 cell apoptosis. Our data showed that TcdA induced apoptosis of NCM460 cells through gC1qR-dependent and mitochondrial dependent pathways.

## Methods

### Chemicals and reagents

Human colonic NCM 460 epithelial cells were purchased from the American Type Culture Collection (ATCC, Manassas, VA). For in vitro experiments, toxin A (TcdA) from *C. difficile* was obtained from Sigma-Aldrich (St. Louis, MO; C3977-2UG). Dulbecco’s Modified Eagle’s Medium (DMEM) powder, 1% L-glutamine, 100 g/ml streptomycin, 10 units/ml penicillin, and fetal bovine serum (FBS) were purchased from the Gibco (Grand Island, NY, USA); Lipofectin transfection reagent was purchased from Invitrogen (Burlington, ON, Canada). Dimethyl sulfoxide (DMSO) and Annexin V-FITC/Propidium Iodide (PI) Flow Cytometry Assay Kit was purchased from Sigma-Aldrich (St. Louis, MO, USA). Small-interfering RNA (siRNA) were synthesised by Wuhan Genesil Biotechnology Co., Ltd. (Wuhan, China). An unrelated gene siRNA was chosen as a negative control. All solvents and chemicals were analytical grade.

### Cell culture

The human colonic NCM 460 epithelial cells were maintained in DMEM medium containing 10% FBS, 2 mM of glutamine, 1% nonessential amino acids, and antibiotics (100 units/ml of penicillin and streptomycin). The cells were cultured in a 5% CO_2_ incubator at 37 °C. The human colonic NCM 460 epithelial cells were maintained to approximately 80% confluence, then treated with a final concentration of 5 ng/ml, 10 ng/ml or 20 ng/ml TcdA for 24 h in a complete medium. Control group (Mock group) were exposed to media only. Toxin A concentrations were selected based on previous data [33].

### Cloning and transfection of full-length gC1qR plasmids

The full-length gC1qR open reading frame (ORF) was subcloned into the pcDNA 3.1 expression plasmid (Invitrogen, Carlsbad, CA). gC1qR sequences were amplified using a specific forward primer which contained a BamHI site; 5′-CCG GTA CCA TGC TGC CTC TGC TGC GCT GCT G-3, and a reverse primer containing a EcoRI site; 5′-CCT CTA GAC TCT GGC TCT TGA CAA AAC TCT TGA GG-3. The PCR product was digested with BamHI and EcoRI and ligated into pcDNA 3.1expression plasmid. The resulting pcDNA3.1-gC1qR vector was then transfected into human colonic NCM 460 epithelial cells.

### gC1qR siRNA-expressing plasmid construction

gC1qR siRNA target sequences as follows: 5′-GCA TCC CAC CAA CAT TTG ATT and 5′-CCG ACG GAG ACA AAG CTT TCT, which were derived from complementary sequences located at exon 3 and the junction between exons 1 and 2 of gC1qR (GenBank™ accession number NM_001212), respectively. The pGenesil-1 vector using eGFP as the reporter gene was purchased from Wuhan Genesil Biotechnology Co., Ltd. The NCM 460 cells were transfected with a mixture containing Lipofectamine, optiMem, and siRNA oligonucleotides (50 μM) according to the manufacturer’s instructions. The gC1qR siRNA-Lipofectamine complex was then added to NCM 460 cells in suspension and plated on 96-well plates (18,000 cells / well). After 48 h, the complete medium was added, along with TcdA (10 ng/ml). The cells were incubated for 24 h, and assays were performed to detect cell viability, cell apoptosis and cell mitochondrial function. At the same time, an unrelated gene siRNA was chosen as a negative control. The sequences as follows: 5′-GCT GAG AGT GAC ATC TTC TCT-3′ or 5′-GGA TCT GCA GTA CTT GCT TCA-3′.

### Cell viability assay 2

The NCM 460 cells viability was assessed by WST-1 assay. Cells were cultured in 96-well plates (1 × 10^5^/well) and treated with 5 ng/ml, 10 ng/ml or 20 ng/ml TcdA. After 24 h of incubation, 10 μL of WST-1 solution (stock solution of 5 mg/mL in PBS) was added to 96-well plates, and the plates were maintained for an additional 4 h at 37 °C. The reducing activity of the cells was examined by treatment with dimethyl sulfoxide (DMSO, 150 μl) prior to reading at an optical density (OD) of 490 nm with an automatic microplate reader (Elx808; BioTek Instruments, Inc., Winooski, VT, USA).

### Detection of apoptotic cells

The NCM 460 cells apoptosis were examined by Annexin V-FITC/ propidium iodide with flow cytometry analysis. The NCM 460 cells were washed and resuspended in binding buffer including 10 mM HEPES, 140 mM NaCl, 2.5 mM CaCl_2_, pH 7.4 for 20 min at room temperature. The cell suspension (1 × 10^6^/well) were mixed with 10 μL of Annexin V-FITC, and incubated for 30 min at room temperature. Then the mix stained with 10 μL of PI solution for additional 10 min on ice. The scatter parameters of the cells were calculated by Coulter EPICS XL flow cytometer (EasyCyte; Guava Technologies).

### Terminal Deoxynucleotidyl Transferase-mediated Deoxyuridine triphosphate Nick-end labeling (TUNNEL) assay

The NCM 460 cells were exposed to the 5 ng/ml, 10 ng/ml or 20 ng/ml TcdA for 24 h in a complete medium, and then fixed with 4% paraformaldehyde for additional 20 min and embedded in paraffin at room temperature. The slices were treated with hydrogen peroxide block for 10 min and incubated with 50 μl of TUNEL reaction mixtures for 60 min at 37 °C in the dark. After being washed twice with PBS, 50 μL of converter-peroxidase were added on the slices and incubated for 40 min at 37 °C, finally, the slices were added to 50 μL of diaminobenzidine substrates for 15 min at 25 °C. After being washed twice with PBS, cells apoptosis level was assessed under a light microscope. Cells with shrunken brown-stained nuclei were considered positive.

### Real-time quantitative polymerase chain reaction (real-time qPCR)

Total RNA was extracted from cultured NCM 460 cells with a Total RNA Extraction Kit (Promega, Beijing, China), 10 μL of RNA was reverse-transcribed into complementary DNA (cDNA) according to protocol provided by the manufacturer (Invitrogen, Carlsbad, CA). gC1qR primer pair: sense, 5′- GCT GCG GCT CGC TGC ACA CCG ACG G − 3′; and antisense, 5′-CTA CTG GCT CTT GAC AAA ACT CTT GAG-3′. β-actin sense: 5′-CGA GCG GGA AAT CGT GCG TGA CAT − 3′; and antisense, 5′-CGT CAT ACT CCT GCT TGC TGA TCC ACA TCT − 3′. Real-time qPCR was performed using a SYBR Green PCR Kit (Invitrogen) on an ABI PRISM 7500 real-time PCR system (Applied Biosystems). The gC1qR mRNA level was determined using the threshold cycle (2^-ΔΔCT^) method [34]. The relative amounts of target gene were normalized to the average of the endogenous control.

### Western blot analysis

The NCM 460 cells were treated with various treatments, and total protein was harvested and lysed in buffer containing 1 mM of EDTA, 0.5% NP-40, 50 mM of Tris-HCl (pH 7.4), 50 mM of NaF, 1% Triton X-100, 1 mM of PMSF, 10% glycerol, 150 mM of NaCl, 1 mM of Na_3_VO_4_, and 1% protease inhibitor cocktail. The equal amount of concentrated proteins were separated using an 10% gradient SDS--polyacrylamide gel running at 100 V for 2 h and transferred onto a polyvinylidene fluoride (PVDF) membrane at 300 mA for 90 min. Non-specific membrane binding sites were blocked in 5% non-fat milk in PBST I for 1 h, and then incubated with primary antibodies specific to gC1qR (1: 1000 dilution, a recombinant rabbit monoclonal antibody, Abcam: ab131284) and actin (1: 2000; ab8227, Abcam) in blocking solution at 37 °C for 2 h. After being washed twice with TBST, the membrane was incubated for 1 h with horseradish peroxidase (HRP)-conjugated secondary antibody (1: 4000; Santa Cruz). The protein band visualization was detected using enhanced chemiluminescence from Cell Signaling Technology (Beverly, MA, USA). The values used for the histogram were normalized to the endogenous control.

### Immunohistochemistry

The cultured NCM 460 cells were trypsinized and pelleted at 1000 X for 20 min at 4 °C. Removing the supernatant, the cells mass were embedded in paraffin and cut into 5 μm and dried at 70 °C for 2 h. According to the standard procedures, the slice was treated with 3% H_2_O_2_ for 25 min in the dark. After being washed twice with PBS, the section were underwent epitope retrieval (5 min 750 W, 15 min 350 W in a microwave) in 0.01 mol/L citrate buffer (pH 6.0). The section was incubated with primary antibodies specific to gC1qR (1: 100 dilution, Abcam: ab131284). After being washed twice with PBS, the section was incubated for 30 min with horseradish peroxidase (HRP)-conjugated secondary antibody (1: 4000; Santa Cruz). After being washed twice with PBS, each section was treated with streptavidin-peroxidase complex, and images were recorded under a confocal laser-scanning microscope (Leica, Germany). Since gC1qR localizes to the mitochondrial matrix, which is expressed in the cytoplasm, cells staining brown in their cytoplasm were considered positive.

### Electron microscope

The cultured NCM 460 cells were digested by trypsin and pelleted at 1000 X for 20 min at 4 °C. After the supernatant was removed, cells were fixed with 2% glutaraldehyde in 0.1 mol/L cacodylate buffer (pH 7.4), the cells mass were post-fixed in 1% OsO4 for 1 h and stained with 1% uranyl acetate for 2 h. Then the cell mass was dehydrated at an acetone series concentration of 50% for 15 min, 70% for 15 min, 80% for 15 min, 90% for 15 min and 100% for 15 min respectively. The mass embedded in Durcupan and sectioned to 60–70 nm thickness. The ultrastructure of NCM 460 cells was examined at 3700 X magnification, and photographs were observed under a Zeiss 10^9^ electron microscope (Carl Zeiss, Oberkochen, Germany).

### Assay of intracellular ROS

Intracellular ROS production was quantified by a ROS assay kit (Beyotime, Shanghai, China). Briefly, at least 1 × 10^5^ NCM 460 cells were incubated with 10 μM final concentration of H_2_DCFDA, followed by incubation for 40 min at 37 °C. After being washed twice with PBS, cells were harvested. ROS level were analyzed with flow cytometry (BD FACSCalibur, San Jose, CA, USA) using 488 nm excitation and 530 nm emission wavelength.

### Measurement of mitochondrial membrane potential (ΔΨm)

Loss of mitochondrial membrane potential (ΔΨm) was examined in NCM 460 cells using the membrane-permeable JC-1 dye (Beyotime). According to the manufacturer’s instructions, NCM 460 cells were loaded with 10 μM JC-1 for 20 min at room temperature. Depolarization of ΔΨm was analyzed by monitoring the fluorescence intensities at the excitation wavelength 485 nm and the emission wavelength 530 nm using fluorescence microscopy.

### Measurement of intracellular ATP levels

The ATP content in NCM 460 cells lysates was detected using an ATP Bioluminescent Cell Assay Kit (S0026, Beyotime, Shanghai, China) according to the manufacturer’s instruction, NCM 460 cells were washed twice in cold PBS buffer, 1 mL of 2% trichloroacetic acid was added into 0.1 mol/L Tris and 2 mmol/L EDTA to stop the luciferase reaction. Cells were removed with a scraper and collected in 1.8-mL Eppendorf centrifuge tubes for 10 min at 15,000 rpm at 4 °C. Supernatants were diluted at 0.1 mol/L Tris–2 mmol/L EDTA (Tris-EDTA) (1: 50), and then ATP was determined and protein was analyzed by pellets. 0.6 mg/mL oligomycin were added to inhibit mitochondrial ATP synthase, and further ATP production was measured. Mitochondrial ATP production was calculated as difference between ATP produced before and after the addition of oligomycin. The absorbance of samples was examined using a TD-20/20 Luminometer (Turner Designs, Sunnyvale, CA, USA). A standard curve of ATP concentrations ranging from 0 to 200 nmol/mL was used in this experiment.

### Statistical analysis

All data are displayed as the mean ± standard deviation (SD). Student’s t test was used to compare the means of two groups. *p*-values less than 0.05 were considered significant (*p < 0.05; ** *p* < 0.01; ^#^ *p* > 0.05). Statistical analysis of the data was performed using SPSS18.0. All experiments were performed in triplicate.

## Supplementary information

**Additional file 1: Figure S1.** gC1qR expression was detected by western blot analysis. The human colonic epithelial cells were transfected with gC1qR vector, empty vector or plain medium (Mock) for 48 h. The expression efficiency of gC1qR protein was analyzed by western blot assay. **p < 0.01, ^#^*p* > 0.05 versus Mock group.

**Additional file 2: Figure S2.** gC1qR expression was detected by western blot analysis. The human colonic epithelial cells were transfected with gC1qR siRNA, negative siRNA or plain medium (Mock) for 48 h. The silencing efficiency of gC1qR gene was analyzed by western blot assay. **p < 0.01, ^#^*p* > 0.05 versus Mock group.

**Additional file 3: Figure S3.** Localization of gC1qR protein expression. Immunofluorescence laser confocal microscopy revealed a large number of C1q-gC1qR complexes anchored on the mitochondrial membrane surface in TcdA-induced human colonic epithelial cell (5200X).

**Additional file 4: Figure S4.** The mitochondrial respiratory chain NDUFS3 and Core 2 protein expression was detected by western blot analysis. The human colonic epithelial cells were transfected with gC1qR siRNA, negative siRNA or plain medium (Mock) for 48 h. The expression of NDUFS3 and Core 2 protein was analyzed by western blot assay. **p < 0.01, ^#^*p* > 0.05 versus Mock group.

**Additional file 5: Figure S5.** The apoptosis of human colonic epithelial cells was detected by flow cytometric analysis. The NCM 460 cells were incubated with TcdA (10 ng/ml) in combination with rotenone (60 μM) (complex I inhibitor) or antimycine A (30 μM) (complex III inhibitor) or transfection with gC1qR siRNA vector. Apoptotic death of NCM 460 cells was examined by flow cytometric analysis. The data are means ± S.D. of three separate experiments performed in triplicate. **p < 0.01 versus TcdA (+) group.

**Additional file 6: Figure S6.** The ATP levels in NCM 460 epithelial cell were assessed. NCM 460 cells were transfected with gC1qR siRNA for 48 h, and then TcdA (10 ng/ml) was added for 24 h. 0.6 mg/mL oligomycin were added to inhibit mitochondrial ATP synthase, and further ATP production was measured. Mitochondrial ATP production was calculated as difference between ATP produced before and after the addition of oligomycin. The data are presented as mean ± S.D. (*n* = 3). **p < 0.01, ^#^*p* > 0.05 versus Mock group.

**Additional file 7: Figure S7.** PDH activity in NCM 460 epithelial cell was assessed. NCM 460 cells were transfected with gC1qR siRNA or negative siRNA for 48 h respectively, and then TcdA (10 ng/ml) was added for 24 h. PDH activity in NCM 460 epithelial cell was assessed. **p < 0.01, ^#^*p* > 0.05 versus TcdA (-), gC1qR siRNA (-) and negative siRNA (-) group; ^△△^*p* < 0.01versus TcdA (+), gC1qR siRNA (-) and negative siRNA (-) group; ^▲▲^p < 0.01 versus TcdA (+), gC1qR siRNA (+) and negative siRNA (-) group.

**Additional file 8: Figure S8.**

**Additional file 9: Figure S9.**

**Additional file 10: Figure S10.** Localization and expression of gC1qR in human colonic epithelial cell lines. NCM 460 cells were transfected with gC1qR siRNA or negative siRNA for 48 h respectively, and then TcdA (10 ng/ml) was added for 24 h. Localization and expression of gC1qR protein was examined using immunohistochemical staining analysis. Cells with brown stained cytoplasm were considered positive.

**Additional file 11: Figure S11.** The apoptosis and ROS generation of human colonic epithelial cells was detected. The NCM 460 cells were transfected with gC1qR siRNA vector in combination with incubation with TcdA (10 ng/ml). (a) Apoptotic death of NCM 460 cells was examined by flow cytometric analysis. (b) Intracellular ROS generation was measured by fluorescence of H_2_DCFDA (Green). The data are means ± S.D. of three separate experiments performed in triplicate. ^#^*p* > 0.05 versus gC1qR siRNA group.

**Additional file 12: Figure S12.** Intracellular TcdA and gC1qR expression. The NCM 460 cells were transfected with gC1qR siRNA vector in combination with incubation with TcdA (10 ng/ml). Intracellular TcdA (blue fluorescence) and gC1qR protein expression (green fluorescence) was indicated by cell immunofluorescence assay.

**Additional file 13: Figure S13.** A brightfield images of the NCM 460 cells . The NCM 460 cells were transfected with gC1qR siRNA vector in combination with incubation with TcdA (10 ng/ml). Representative electron microscopy images, a brightfield picture showing cells round up. (Red arrows). Scale bar: 100 μm.

**Additional file 14: Figure S14.** Intracellular TcdA level was detected by western blot analysis. The NCM 460 cells were transfected with gC1qR siRNA vector in combination with incubation with TcdA (10 ng/ml). Intracellular TcdA level was analyzed by western blot assay. **p < 0.01 versus Tcd A group.

**Additional file 15: Figure S15.** Apoptotic death of NCM 460 cells. The NCM 460 cells were transfected with gC1qR siRNA vector or treated with α-lipoic acid in combination with incubation with TcdA (10 ng/ml). Apoptotic death of NCM 460 cells was examined by flow cytometric analysis. The data are means ± S.D. of three separate experiments performed in triplicate. **p < 0.01 versus Tcd A group.

## Data Availability

All data generated or analyzed during this study are included in this published article.

## Abbreviations

*C. difficile*: *Clostridioides difficile*
TcdA: *C. difficile* toxin A
gC1qR: Globular heads receptor of C1q
PCR: Real-time polymerase chain reaction
WST-1: Water-soluble tetrazolium salt
TUNEL): Terminal deoxynucleotidyl transferase-mediated deoxyuridine triphosphate nick-end labeling
ROS: Reactive oxygen species
ΔΨm: Mitochondrial membrane potential
