# Supplementary figures and images for: The role of the globular heads of the C1q receptor in TcdA-induced human colonic epithelial cell apoptosis via a mitochondria-dependent pathway
Source: BMC Microbiol. 2020 Sep 2;20:274. doi: 10.1186/s12866-020-01958-6 (PMC7465811; doi:10.1186/s12866-020-01958-6)

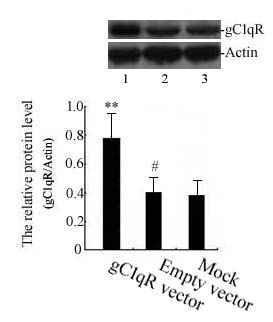

Supplement: Supplementary file 1 — Additional file 1: Figure S1. gC1qR expression was detected by western blot analysis. The human colonic epithelial cells were transfected with gC1qR vector, empty vector or plain medium (Mock) for 48 h. The expression efficiency of gC1qR protein was analyzed by western blot assay. **p < 0.01, #p > 0.05 versus Mock group. [file 12866_2020_1958_MOESM1_ESM.jpg]

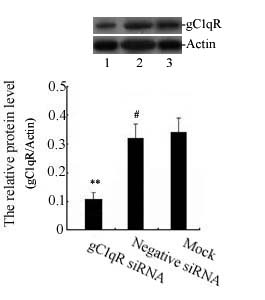

Supplement: Supplementary file 2 — Additional file 2: Figure S2. gC1qR expression was detected by western blot analysis. The human colonic epithelial cells were transfected with gC1qR siRNA, negative siRNA or plain medium (Mock) for 48 h. The silencing efficiency of gC1qR gene was analyzed by western blot assay. **p < 0.01, #p > 0.05 versus Mock group. [file 12866_2020_1958_MOESM2_ESM.jpg]

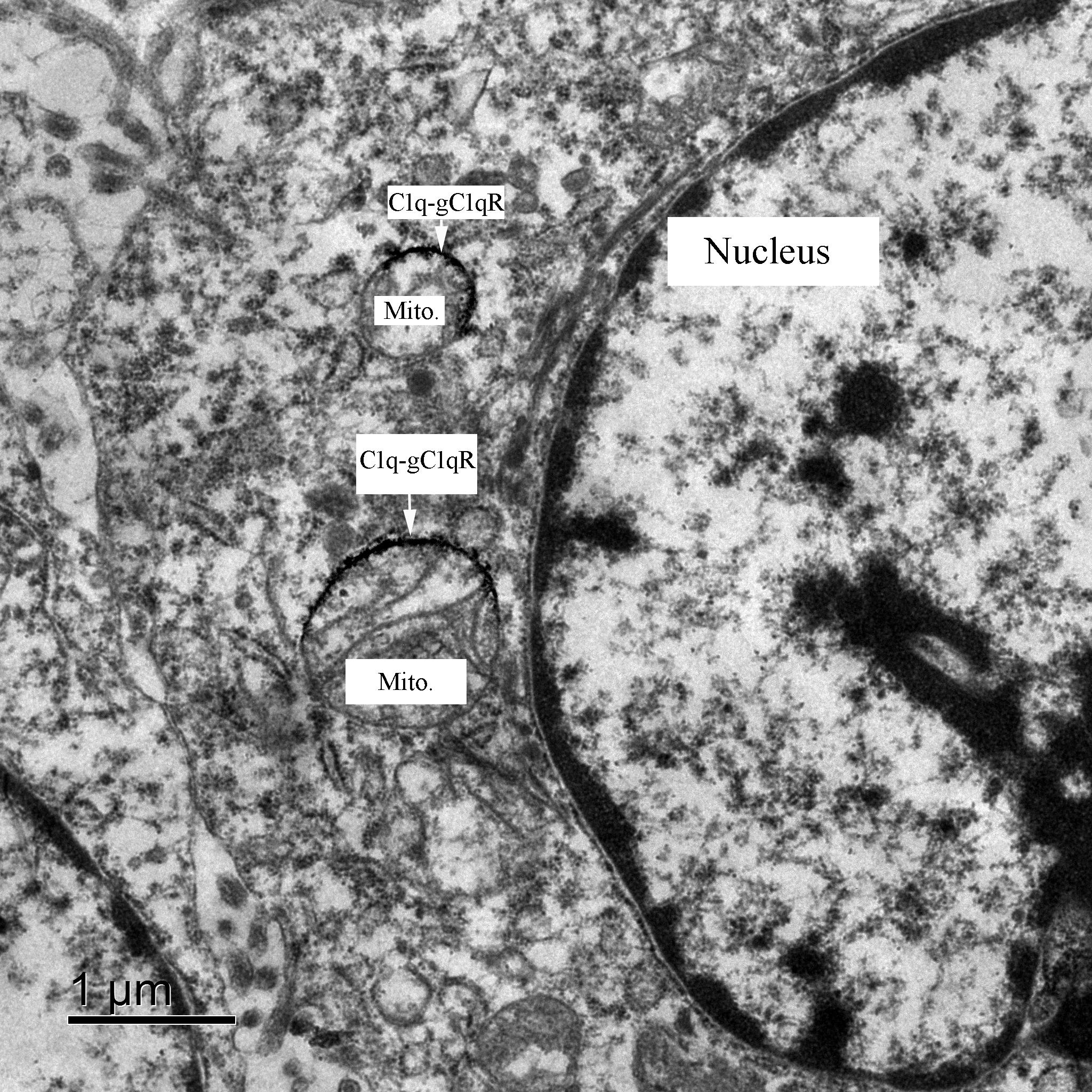

Supplement: Supplementary file 3 — Additional file 3: Figure S3. Localization of gC1qR protein expression. Immunofluorescence laser confocal microscopy revealed a large number of C1q-gC1qR complexes anchored on the mitochondrial membrane surface in TcdA-induced human colonic epithelial cell (5200X). [file 12866_2020_1958_MOESM3_ESM.tif]

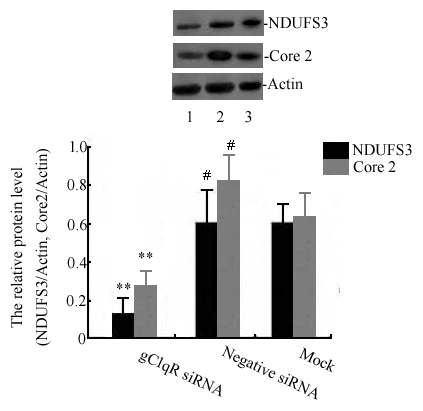

Supplement: Supplementary file 4 — Additional file 4: Figure S4. The mitochondrial respiratory chain NDUFS3 and Core 2 protein expression was detected by western blot analysis. The human colonic epithelial cells were transfected with gC1qR siRNA, negative siRNA or plain medium (Mock) for 48 h. The expression of NDUFS3 and Core 2 protein was analyzed by western blot assay. **p < 0.01, #p > 0.05 versus Mock group. [file 12866_2020_1958_MOESM4_ESM.tif]

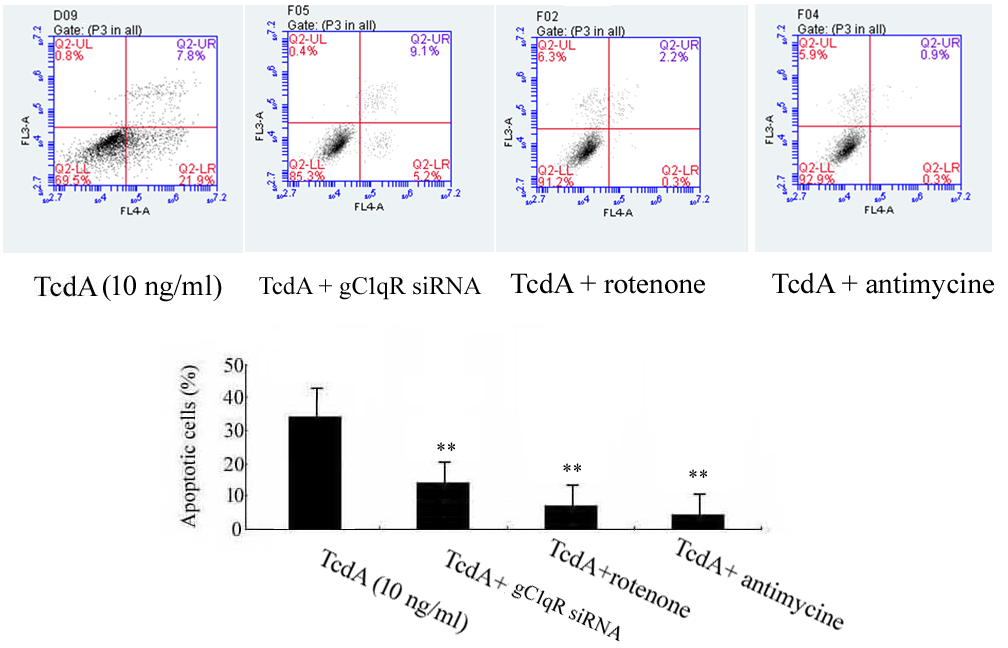

Supplement: Supplementary file 5 — Additional file 5: Figure S5. The apoptosis of human colonic epithelial cells was detected by flow cytometric analysis. The NCM 460 cells were incubated with TcdA (10 ng/ml) in combination with rotenone (60 μM) (complex I inhibitor) or antimycine A (30 μM) (complex III inhibitor) or transfection with gC1qR siRNA vector. Apoptotic death of NCM 460 cells was examined by flow cytometric analysis. The data are means ± S.D. of three separate experiments performed in triplicate. **p < 0.01 versus TcdA (+) group. [file 12866_2020_1958_MOESM5_ESM.tif]

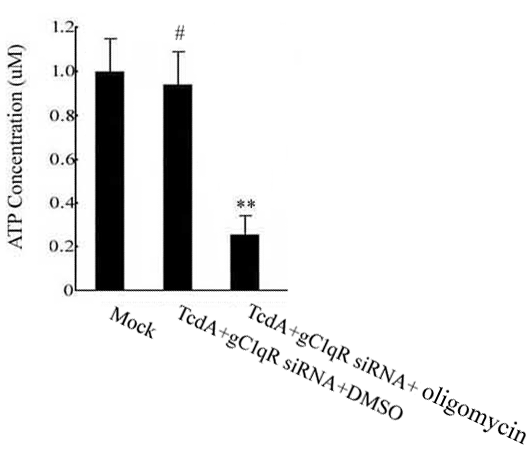

Supplement: Supplementary file 6 — Additional file 6: Figure S6. The ATP levels in NCM 460 epithelial cell were assessed. NCM 460 cells were transfected with gC1qR siRNA for 48 h, and then TcdA (10 ng/ml) was added for 24 h. 0.6 mg/mL oligomycin were added to inhibit mitochondrial ATP synthase, and further ATP production was measured. Mitochondrial ATP production was calculated as difference between ATP produced before and after the addition of oligomycin. The data are presented as mean ± S.D. (n = 3). **p < 0.01, #p > 0.05 versus Mock group. [file 12866_2020_1958_MOESM6_ESM.tif]

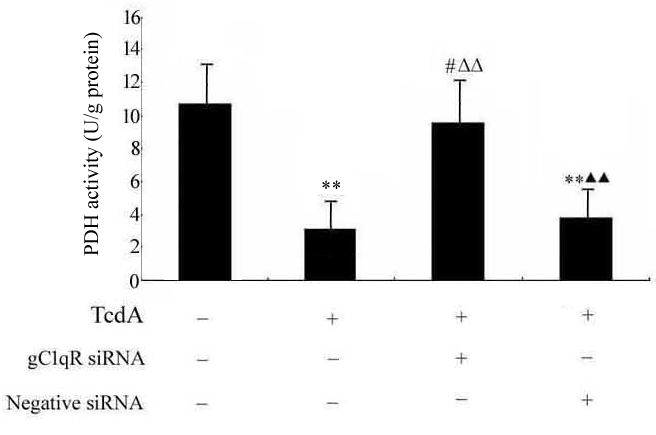

Supplement: Supplementary file 7 — Additional file 7: Figure S7. PDH activity in NCM 460 epithelial cell was assessed. NCM 460 cells were transfected with gC1qR siRNA or negative siRNA for 48 h respectively, and then TcdA (10 ng/ml) was added for 24 h. PDH activity in NCM 460 epithelial cell was assessed. **p < 0.01, #p > 0.05 versus TcdA (-), gC1qR siRNA (-) and negative siRNA (-) group; △△p < 0.01versus TcdA (+), gC1qR siRNA (-) and negative siRNA (-) group; ▲▲p < 0.01 versus TcdA (+), gC1qR siRNA (+) and negative siRNA (-) group. [file 12866_2020_1958_MOESM7_ESM.tif]

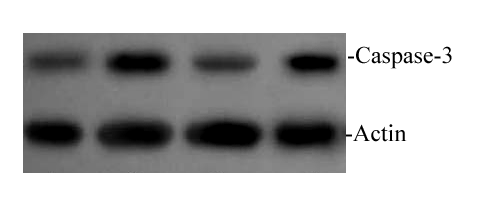

Supplement: Supplementary file 8 — Additional file 8: Figure S8. [file 12866_2020_1958_MOESM8_ESM.tif]

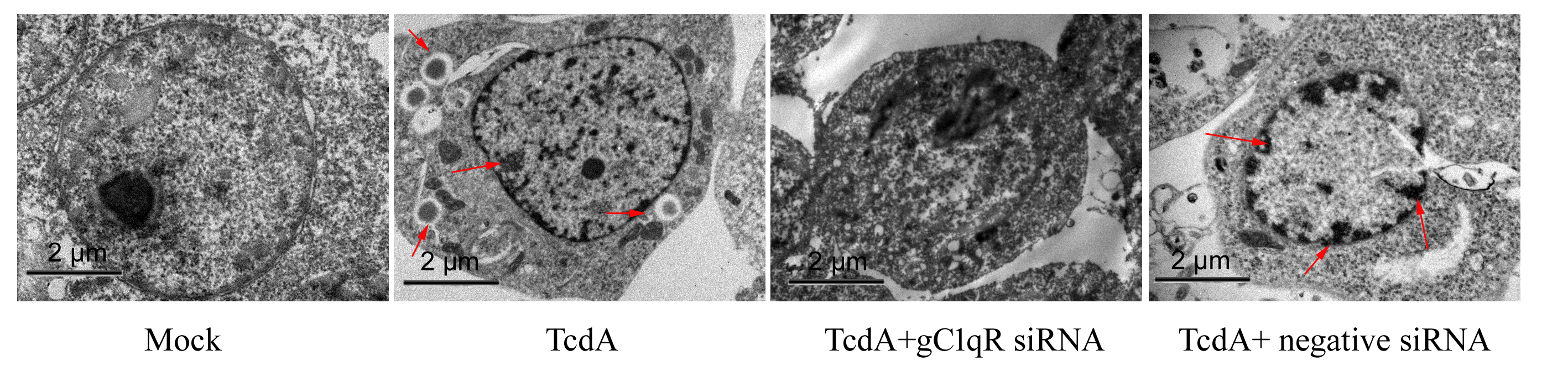

Supplement: Supplementary file 9 — Additional file 9: Figure S9. [file 12866_2020_1958_MOESM9_ESM.tif]

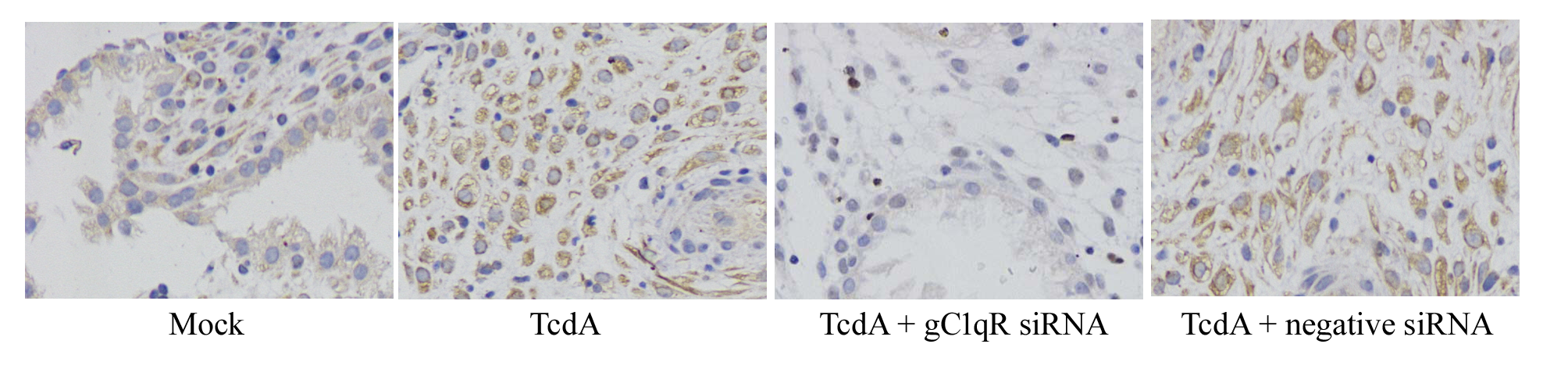

Supplement: Supplementary file 10 — Additional file 10: Figure S10. Localization and expression of gC1qR in human colonic epithelial cell lines. NCM 460 cells were transfected with gC1qR siRNA or negative siRNA for 48 h respectively, and then TcdA (10 ng/ml) was added for 24 h. Localization and expression of gC1qR protein was examined using immunohistochemical staining analysis. Cells with brown stained cytoplasm were considered positive. [file 12866_2020_1958_MOESM10_ESM.tif]

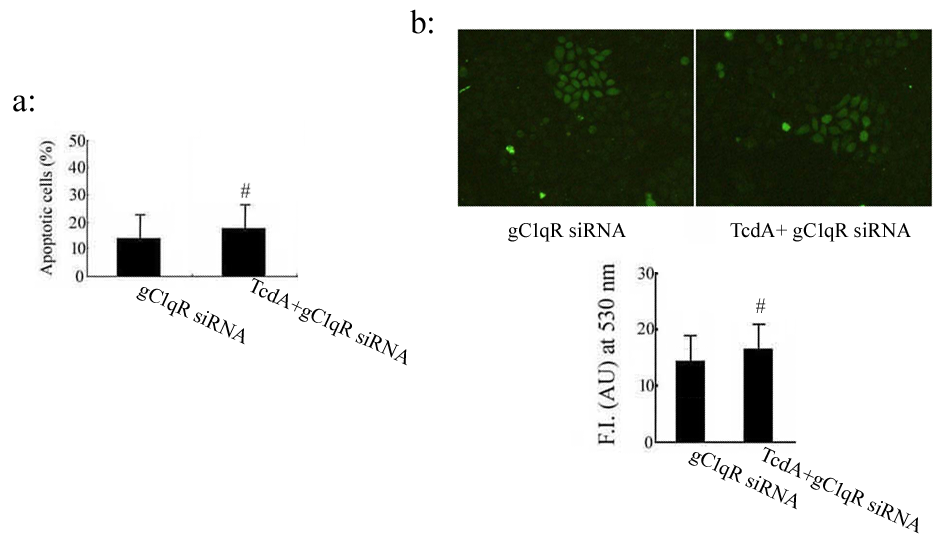

Supplement: Supplementary file 11 — Additional file 11: Figure S11. The apoptosis and ROS generation of human colonic epithelial cells was detected. The NCM 460 cells were transfected with gC1qR siRNA vector in combination with incubation with TcdA (10 ng/ml). (a) Apoptotic death of NCM 460 cells was examined by flow cytometric analysis. (b) Intracellular ROS generation was measured by fluorescence of H2DCFDA (Green). The data are means ± S.D. of three separate experiments performed in triplicate. #p > 0.05 versus gC1qR siRNA group. [file 12866_2020_1958_MOESM11_ESM.tif]

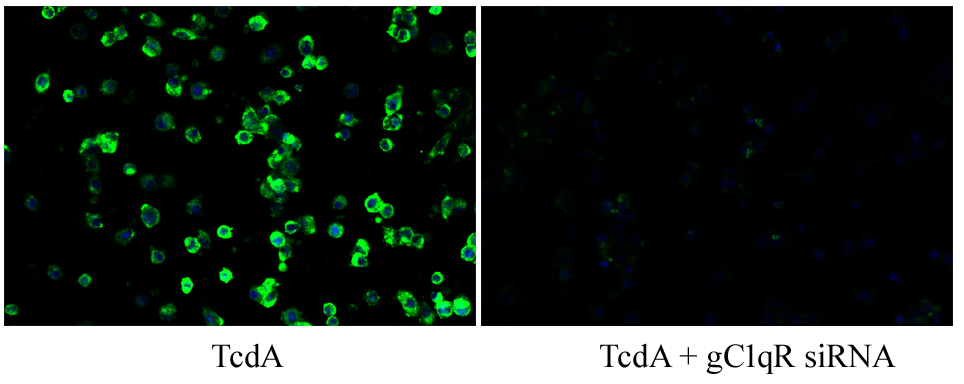

Supplement: Supplementary file 12 — Additional file 12: Figure S12. Intracellular TcdA and gC1qR expression. The NCM 460 cells were transfected with gC1qR siRNA vector in combination with incubation with TcdA (10 ng/ml). Intracellular TcdA (blue fluorescence) and gC1qR protein expression (green fluorescence) was indicated by cell immunofluorescence assay. [file 12866_2020_1958_MOESM12_ESM.tif]

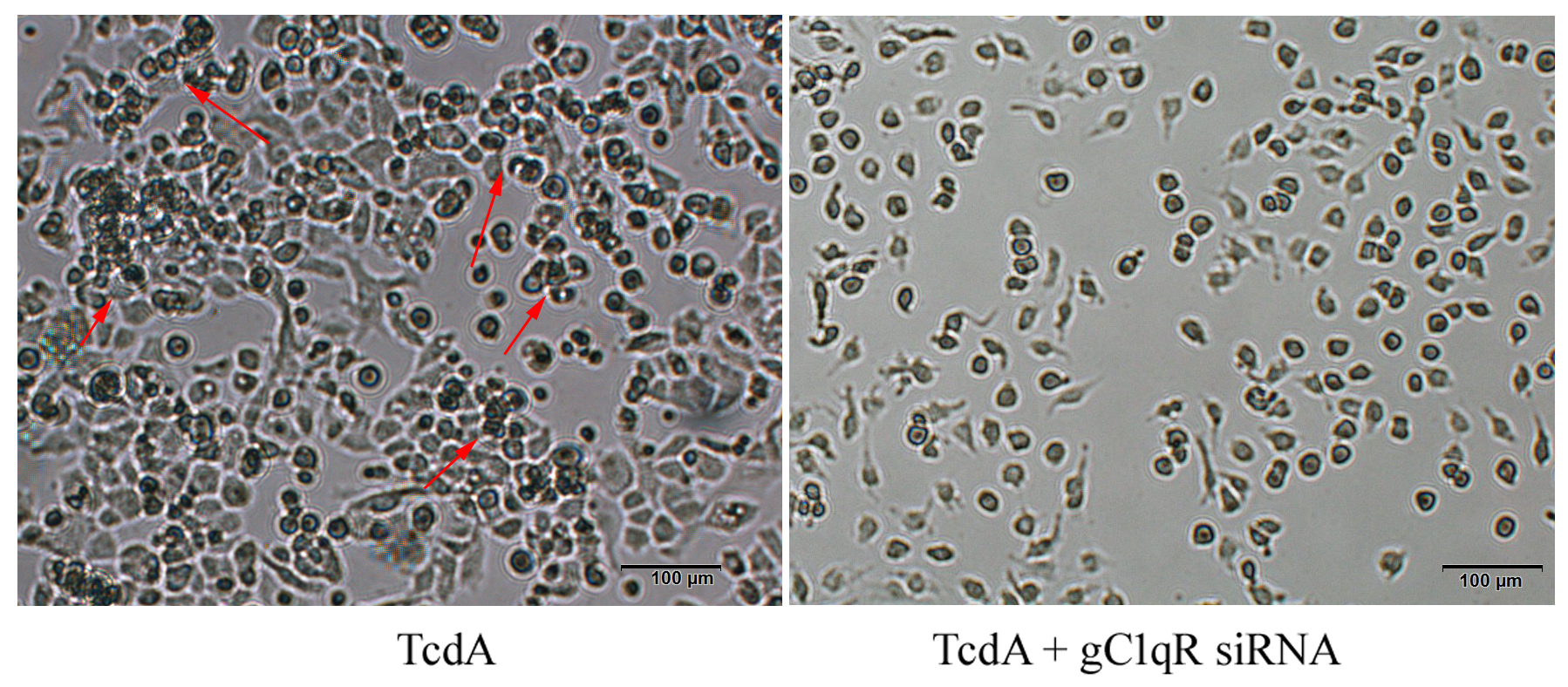

Supplement: Supplementary file 13 — Additional file 13: Figure S13. A brightfield images of the NCM 460 cells . The NCM 460 cells were transfected with gC1qR siRNA vector in combination with incubation with TcdA (10 ng/ml). Representative electron microscopy images, a brightfield picture showing cells round up. (Red arrows). Scale bar: 100 μm. [file 12866_2020_1958_MOESM13_ESM.tif]

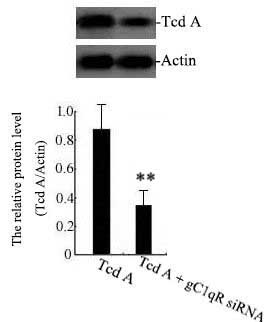

Supplement: Supplementary file 14 — Additional file 14: Figure S14. Intracellular TcdA level was detected by western blot analysis. The NCM 460 cells were transfected with gC1qR siRNA vector in combination with incubation with TcdA (10 ng/ml). Intracellular TcdA level was analyzed by western blot assay. **p < 0.01 versus Tcd A group. [file 12866_2020_1958_MOESM14_ESM.jpg]

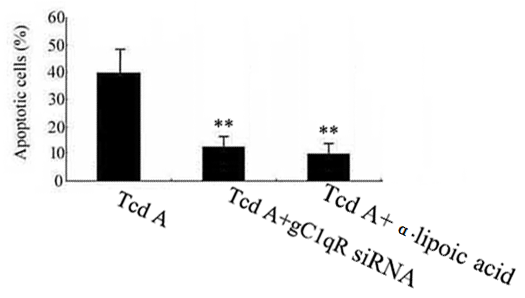

Supplement: Supplementary file 15 — Additional file 15: Figure S15. Apoptotic death of NCM 460 cells. The NCM 460 cells were transfected with gC1qR siRNA vector or treated with α-lipoic acid in combination with incubation with TcdA (10 ng/ml). Apoptotic death of NCM 460 cells was examined by flow cytometric analysis. The data are means ± S.D. of three separate experiments performed in triplicate. **p < 0.01 versus Tcd A group. [file 12866_2020_1958_MOESM15_ESM.tif]
